# Justificativa e Desenho do Ensaio Clínico Randomizado COVID-19 Outpatient Prevention Evaluation (COPE - Coalition V): Hidroxicloroquina vs. Placebo em Pacientes Não Hospitalizados

**DOI:** 10.36660/abc.20210832

**Published:** 2022-02-14

**Authors:** Haliton Alves de Oliveira, Cleusa P. Ferri, Icaro Boszczowski, Gustavo B. F. Oliveira, Alexandre B. Cavalcanti, Regis G. Rosa, Renato D. Lopes, Luciano C. P. Azevedo, Viviane C. Veiga, Otavio Berwanger, Álvaro Avezum

**Affiliations:** 1 Centro Internacional de Pesquisa Hospital Alemão Oswaldo Cruz São Paulo SP Brasil Centro Internacional de Pesquisa, Hospital Alemão Oswaldo Cruz,São Paulo, SP – Brasil; 2 Instituto de Pesquisa HCor São Paulo SP Brasil Instituto de Pesquisa HCor,São Paulo, SP – Brasil; 3 Brazilian Research in Intensive Care Network São Paulo SP Brasil Brazilian Research in Intensive Care Network (BRICnet),São Paulo, SP – Brasil; 4 Hospital Moinhos de Vento Porto Alegre PR Brasil Hospital Moinhos de Vento,Porto Alegre, PR – Brasil; 5 Brazilian Clinical Research Institute São Paulo SP Brasil Brazilian Clinical Research Institute (BCRI),São Paulo, SP – Brasil; 6 Duke University Medical Center Duke Clinical Research Institute Durham EUA Duke University Medical Center – Duke Clinical Research Institute,Durham – EUA; 7 Instituto de Ensino e Pesquisa do Hospital Sírio-Libanês São Paulo SP Brasil Instituto de Ensino e Pesquisa do Hospital Sírio-Libanês,São Paulo, SP – Brasil; 8 Beneficência Portuguesa de São Paulo São Paulo SP Brasil Beneficência Portuguesa de São Paulo,São Paulo, SP – Brasil; 9 Academic Research Organization Hospital Israelita Albert Einstein São Paulo SP Brasil Academic Research Organization, Hospital Israelita Albert Einstein,São Paulo, SP – Brasil

**Keywords:** COVID-19, SARS-CoV-2, Hidroxicloroquina, Ensaio Clínico Controlado Aleatório

## Abstract

**Fundamento:**

Apesar da necessidade de opções terapêuticas específicas para a doença do coronavírus 2019 (covid-19), ainda não há evidências da eficácia de tratamentos específicos no contexto ambulatorial. Há poucos estudos randomizados que avaliam a hidroxicloroquina (HCQ) em pacientes não hospitalizados. Esses estudos não indicaram benefício com o uso da HCQ; no entanto, avaliaram desfechos primários diferentes e apresentaram vieses importantes na avaliação dos desfechos.

**Objetivo:**

Investigar se a HCQ possui o potencial de prevenir hospitalizações por covid-19 quando comparada ao placebo correspondente.

**Métodos:**

O estudo COVID-19 Outpatient Prevention Evaluation (COPE) é um ensaio clínico randomizado, pragmático, duplo-cego, multicêntrico e controlado por placebo que avalia o uso da HCQ (800 mg no dia 1 e 400 mg do dia 2 ao dia 7) ou placebo correspondente na prevenção de hospitalizações por covid-19 em casos precoces confirmados ou suspeitos de pacientes não hospitalizados. Os critérios de inclusão são adultos (≥ 18 anos) que procuraram atendimento médico com sintomas leves de covid-19, com randomização ≤ 7 dias após o início dos sintomas, sem indicação de hospitalização na triagem do estudo e com pelo menos um fator de risco para complicações (> 65 anos, hipertensão, diabetes melito, asma, doença pulmonar obstrutiva crônica ou outras doenças pulmonares crônicas, tabagismo, imunossupressão ou obesidade). Todos os testes de hipótese serão bilaterais. Um valor de p < 0,05 será considerado estatisticamente significativo em todas as análises. Clinicaltrials.gov: NCT04466540.

**Resultados:**

Os desfechos clínicos serão avaliados centralmente por um comitê de eventos clínicos independente cegado para a alocação dos grupos de tratamento. O desfecho primário de eficácia será avaliado de acordo com o princípio da intenção de tratar.

**Conclusão:**

Este estudo apresenta o potencial de responder de forma confiável a questão científica do uso da HCQ em pacientes ambulatoriais com covid-19. Do nosso conhecimento, este é o maior estudo avaliando o uso de HCQ em indivíduos com covid-19 não hospitalizados.

## Introdução

Em dezembro de 2019, um grupo de pacientes com pneumonia de causa desconhecida foi identificado em Wuhan, na província de Hubei, na China.^1^ O sequenciamento de alto rendimento de amostras do trato respiratório inferior indicou a presença de um novo coronavírus, chamado novo coronavírus 2019 ou, mais recentemente, coronavírus ^2^ causador da síndrome respiratório aguda grave (SARS-CoV-2), que causa uma condição clínica complicada que afeta a função pulmonar (denominada doença do coronavírus 2019, ou covid-19), a qual ainda não havia sido detectada em seres humanos ou animais.^1^

Apesar da necessidade de terapias específicas para a covid-19, não há evidências claras da efetividade de nenhum tratamento no contexto ambulatorial. Dessa forma, a avaliação de opções terapêuticas, como agentes farmacológicos com propriedades antivirais, é essencial para reduzir o risco de deterioração clínica, hospitalização, necessidade de ventilação mecânica e morte, especialmente na fase inicial da covid-19 no contexto ambulatorial. Atualmente, há alguns ensaios clínicos randomizados (ECRs) avaliando o efeito da cloroquina/hidroxicloroquina (HCQ) em pacientes não hospitalizados. No contexto da profilaxia pré e pós-exposição, os estudos clínicos não identificaram benefícios em relação à taxa de infecção da covid-19,^5^ enquanto outros estudos identificaram um aumento na ocorrência de efeitos adversos em pacientes que receberam cloroquina/HCQ.^6 , 7^ Contudo, é importante ressaltar que esses estudos continham vieses significativos e, quando considerados em conjunto, apresentaram heterogeneidade significativa de resultados devido a diferenças nos regimes de dosagem, critérios de inclusão e desfechos primários.

Considerando os casos de covid-19 não hospitalizados, os ECRs não encontraram diferenças significativas na taxa de hospitalização ao comparar HCQ e placebo^10^ ou cuidado usual.^11^ Além disso, alguns ECRs não identificaram benefício em relação à cura ou redução da carga viral ao comparar HCQ e placebo^12^ ou cuidado usual.^10^ Alguns ECRs relataram um aumento na ocorrência de eventos adversos em pacientes que receberam HCQ.^10 , 11^ Portanto, são necessários estudos mais abrangentes, com melhor rigor metodológico.

O principal objetivo do presente estudo é investigar se o tratamento precoce com HCQ diminui o risco de hospitalização (desfecho primário de eficácia) por complicações clínicas relacionadas à covid-19 em 30 dias após a randomização.

## Métodos

### Desenho do estudo

**Figura 1:**
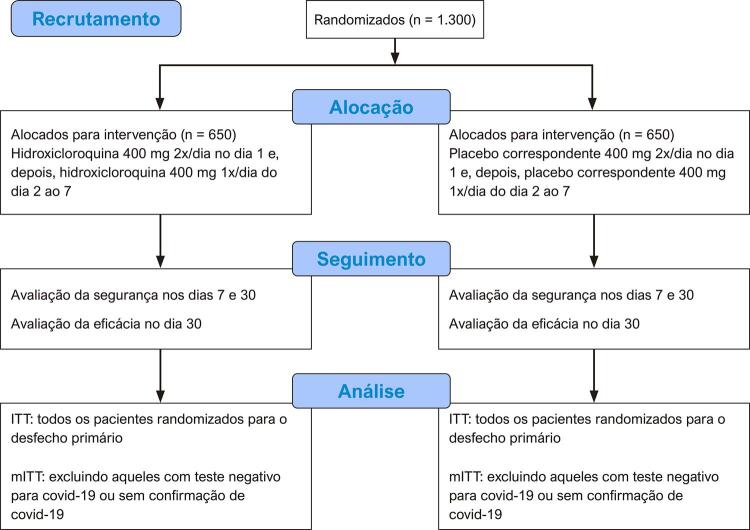
–Diagrama CONSORT mostrando o fluxo de trabalho e o recrutamento planejado para o estudo. ITT: intenção de tratar; mITT: intenção de tratar modificada.

Este é um ensaio clínico randomizado, pragmático, duplo-cego, multicêntrico e controlado por placebo. Foi realizada alocação na proporção 1:1 para avaliar as potenciais propriedades antivirais do tratamento precoce com HCQ (800 mg VO no dia 1 e 400 mg VO do dia 2 ao dia 7) em comparação com placebo correspondente na prevenção de hospitalizações por complicações relacionadas à covid-19 em pacientes não hospitalizados com suspeita ou confirmação de covid-19. O fluxo de trabalho planejado é apresentado na Figura 1 . O presente estudo segue os Standard Protocol Items: Recommendations for Interventional Trials (SPIRIT) (Tabela Suplementar 1).

### Local do estudo

Este estudo será conduzido em 56 centros de diferentes regiões geográficas do Brasil. Os centros são serviços de saúde ambulatoriais privados e públicos que receberam aprovação para participar do estudo após avaliação de viabilidade favorável, conformidade com as boas práticas clínicas e aprovação do comitê de ética.

### Objetivo primário

Objetivamos investigar se o tratamento com HCQ está associado a uma redução na necessidade de hospitalização por complicações clínicas relacionadas à covid-19 suspeita ou confirmada em 30 dias após a randomização no contexto ambulatorial. A hospitalização é definida como um período de permanência hospitalar ≥ 24 horas ou pelo menos mais um dia de hospitalização adjudicada. Os critérios de hospitalização seguirão a prática clínica local de cada centro participante.

### Objetivos secundários

Avaliar o efeito do tratamento com HCQ comparado ao placebo em pacientes ambulatoriais com suspeita ou confirmação de covid-19, aos 30 dias de seguimento, em relação aos seguintes desfechos:

1. Asma não controlada após ≥ 5 dias desde o início do medicamento: resposta afirmativa em três ou quatro itens do questionário Global Initiative for Asthma, descrito na Tabela Suplementar 2;
2. Pneumonia: definida por critérios clínico-radiológicos, incluindo histórico de tosse e a presença de um ou mais dos seguintes sinais/sintomas: escarro, dispneia, dor no peito, suor ou febre (> 37,8o C) + tomografia computadorizada do tórax com opacidade em vidro fosco uni ou bilateral, consolidações focais ou opacidades mistas (incluindo sinal do halo invertido);
3. Otite média: definida por critérios clínicos de febre (> 37,8o C) e otalgia + protuberância da membrana timpânica;
4. Tempo até a resolução da febre: o dia 0 da resolução da febre será definido como o primeiro dia sem febre (< 37,5o C) após a inclusão no estudo, seguido por pelo menos 2 dias consecutivos. A temperatura será obtida através de relato do participante no diário do paciente;
5. Tempo até a melhora dos sintomas respiratórios (tosse, coriza);
6. Hospitalização em unidade de terapia intensiva (UTI): admissão na UTI por complicações clínicas relacionadas à covid-19;
7. Necessidade de intubação orotraqueal: necessidade clínica conforme a avaliação do médico assistente;
8. Duração da ventilação mecânica: número de dias na ventilação mecânica até a extubação ou morte;
9. Mortalidade: morte por qualquer causa que ocorra em 30 dias após a randomização;

A segurança clínica também será avaliada em 30 dias após a randomização:

1. Hipoglicemia: alteração na frequência de episódios em pacientes diabéticos em uso de hipoglicemiantes, percebida por sinais ou sintomas clínicos ou medida em dispositivos de glicemia capilar ou venosa;
2. Palpitações: autopercepção de batimentos cardíacos que poderiam ser diagnosticados como arritmias em pacientes sem histórico conhecido de intervalo QTc prolongado ou doença cardíaca preexistente;
3. Acuidade visual reduzida: alteração na acuidade visual ou diagnóstico recente de retinopatia não documentada anteriormente;
4. Diarreia: alteração no hábito intestinal com três ou mais episódios diarreicos por dia durante o uso de HCQ e 3 dias após o término do tratamento;
5. Anorexia: alteração no apetite durante o uso de HCQ e 3 dias após o término do tratamento;
6. Labilidade emocional: percepção de alteração na labilidade emocional (oscilações de humor) durante o uso de HCQ e 3 dias após o término do tratamento.

### Objetivos exploratórios

Avaliar o efeito do tratamento com HCQ comparado ao placebo em pacientes ambulatoriais com suspeita ou confirmação de covid-19 em relação aos seguintes desfechos:

1. Tempo até a hospitalização após a randomização;
2. Avaliação do estado clínico do paciente no momento da hospitalização.

### Critérios de elegibilidade

Definição de caso de covid-19:

- A avaliação clínica inicial dos pacientes e a triagem de elegibilidade para o estudo serão realizadas com base na classificação de casos confirmados e suspeitos, desenvolvida de acordo com as diretrizes do Ministério da Saúde e as recomendações da Organização Mundial da Saúde^13 , 14^ sobre a definição de casos, adaptada para o contexto ambulatorial ( Tabela 1 ). Os critérios de exclusão e inclusão estão descritos na Tabela 2 , incluindo aqueles relacionados à segurança cardiovascular, visto que a HCQ pode prolongar o intervalo QTc.^15^
  **Tabela 1:** Definição de caso de covid-19 para o estudo COPE (COALITION V), adaptado de acordo com as diretrizes do Ministério da Saúde e da Organização Mundial da Saúde

Caso de covid-19	Definição
Confirmado	Indivíduos com confirmação laboratorial de covid-19 (detecção por RT-PCR do vírus SARS-CoV-2), com amostra coletada preferencialmente entre o 4º e o 7º dia desde o início dos sintomas por swabs nasofaríngeos/orofaríngeos, independentemente dos sinais e sintomas.
	Teste imunológico (teste rápido ou sorologia clássica para detecção de anticorpos IgM/IgG) em amostra coletada a partir do 7º dia desde o início dos sintomas e analisada por método validado.
Suspeito	Pacientes que atendem a pelo menos um dos seguintes critérios*:
	Pacientes com doença respiratória aguda (febre E pelo menos um sinal/sintoma de doença respiratória, como tosse ou dispneia) E histórico de viagem ou residência em local com relato de transmissão comunitária de covid-19 nos 14 dias anteriores ao início dos sintomas;
	Pacientes com doença respiratória aguda E contato com um caso confirmado ou provável (com doença respiratória aguda sem confirmação laboratorial) de covid-19 nos 14 dias anteriores ao início dos sintomas.

* Dependendo do estado clínico do paciente, esses critérios podem ser complementados por achados radiológicos (infiltração intersticial na radiografia do tórax e/ou opacidade em vidro fosco na tomografia computadorizada do pulmão). É importante ressaltar que a maioria dos pacientes a serem tratados apresentarão sintomas leves, sem indicação clínica para exame de imagem.
  **Tabela 2:** Critérios de inclusão e exclusão

Critérios gerais	
	Adultos (> 18 anos) que procuraram atendimento médico com suspeita ou confirmação de covid-19 ≤ 7 dias após o início dos sintomas, com sintomas leves, sem indicação clara de hospitalização e pelo menos um dos seguintes fatores de risco para complicações clínicas:
**Critérios de inclusão**	
1.	Idade > 65 anos;
2.	Hipertensão;
3.	Diabetes melito;
4.	Asma;
5.	Doença pulmonar obstrutiva crônica ou outras doenças pulmonares crônicas;
6.	Tabagismo;
7.	Imunossupressão;
8.	Obesidade (definida como índice de massa corporal ≥ 30 kg/m2).
**Critérios de exclusão**	
1.	Hospitalização imediata após o primeiro atendimento médico;
2.	Teste positivo para influenza no primeiro atendimento médico;
3.	Hipersensibilidade conhecida à hidroxicloroquina/cloroquina;
4.	Diagnóstico prévio de retinopatia ou degeneração macular;
5.	Diagnóstico prévio de síndrome do QT longo, histórico de morte súbita em parentes próximos (pais e irmãos), insuficiência cardíaca descompensada, doença arterial coronariana instável, uso de medicamentos antiarrítmicos ou outros medicamentos que possam aumentar a biodisponibilidade ou o efeito da hidroxicloroquina;
6.	Evidência de doença hepática conhecida, relatada pelo paciente;
7.	Evidência de doença renal crônica conhecida, relatada pelo paciente;
8.	Pacientes com pancreatite;
9.	Eletrocardiograma basal com intervalo QTc ≥ 480 ms;
10.	Uso crônico de hidroxicloroquina/cloroquina por outros motivos;
11.	Gravidez.

### Método de randomização e alocação oculta

A randomização (1:1) será gerada por um software *on-line* e realizada em blocos de permutação de tamanho 8. A ocultação da lista de randomização é mantida por meio de um sistema de randomização centralizado, automatizado e disponível 24 horas *on-line* .

### Cegamento

Os pacientes, pesquisadores e profissionais de saúde serão cegados para a alocação dos medicamentos. Os desfechos clínicos serão avaliados de forma cega pelo Comitê de Julgamento de Eventos Clínicos.

### Intervenções do estudo

Os dois braços do estudo receberão cuidado usual de acordo com a prática local, que basicamente consiste em recomendações gerais e medicação para alívio dos sintomas. As medidas de apoio e cuidado padrão são definidas como quaisquer tratamentos além dos medicamentos utilizados no estudo necessários para o cuidado do paciente com covid-19, a critério do médico assistente.

Os pacientes do grupo HCQ receberão uma dose de 400 mg 2x/dia no primeiro dia e uma dose de 400 mg 1x/dia do segundo ao sétimo dia. Os pacientes do grupo placebo receberão o mesmo regime de administração.

### Avaliação dos desfechos e seguimento

Serão realizados dois contatos por telefone com os participantes (7 e 30 dias) para avaliar a adesão, os sintomas e a necessidade de atendimento médico para detectar uma possível progressão da doença ou a presença de eventos adversos causados pelo tratamento.

### Interrupção do tratamento

Caso sejam confirmados testes negativos para covid-19 em pacientes com suspeita de infecção, o seguinte procedimento será realizado:

- Quando o teste para SARS-CoV-2 (reação da transcriptase reversa seguida pela reação em cadeia da polimerase/reverse *transcription polymerase chain reaction* , RT-PCR) for realizado no hospital em que o paciente foi randomizado, o pesquisador responsável do centro deverá obter essa informação e compartilhá-la com o centro coordenador (Centro Internacional de Pesquisa do Hospital Alemão Oswaldo Cruz). O paciente será aconselhado a interromper o tratamento e continuará sendo acompanhado até o fim do seguimento de 30 dias.
- Quando o teste para SARS-CoV-2 (RT-PCR) for realizado em outro laboratório, o participante deverá entrar em contato com o centro em que foi randomizado, o qual, então, deverá informar o centro coordenador. O paciente será aconselhado a interromper o tratamento ou não, conforme apropriado, e continuará sendo acompanhado por até 30 dias.

O estudo será interrompido caso sejam observados benefícios claros da intervenção no desfecho primário ou um aumento na frequência de eventos adversos graves. O Comitê de Monitoramento de Segurança de Dados ( *Data Safety Monitoring Board* , DSMB) irá monitorar atentamente qualquer ocorrência de eventos adversos imprevistos ou graves para, se necessário, recomendar o encerramento do estudo para garantir a segurança dos pacientes.

### Relato e manejo de eventos adversos

Neste protocolo, os eventos adversos não são considerados um desfecho do estudo, exceto aqueles classificados como graves (hospitalização por covid-19 e morte).

A coleta ativa dos eventos adversos ocorrerá a partir do momento em que o participante assinar o Termo de Consentimento Livre e Esclarecido (TCLE). As informações coletadas devem incluir os dados do histórico clínico do paciente e as comorbidades, o diagnóstico do evento (com base em sinais e sintomas), a classificação da gravidade, a data de início, a definição da probabilidade de relação causal, bem como a causa do evento de acordo com o pesquisador, a decisão médica, a evolução do paciente em relação aos desfechos adversos, os critérios utilizados para classificar a gravidade do evento e a data de término.

### Relato e julgamento dos desfechos

O desfecho primário será avaliado por médicos com experiência prévia e atual na validação de eventos clínicos com base em padrões internacionais. Hospitalizações em 30 dias por causas relacionadas à covid-19 serão documentadas pela equipe médica. As informações serão coletadas para análise pelo Comitê de Julgamento de Eventos Clínicos, sob alocação confidencial (cegado para a avaliação de eventos clínicos), conforme critérios padronizados. O DSMB avaliará os efeitos da HCQ em comparação ao tratamento com placebo para a medida de desfecho primário (hospitalização em 30 dias) e para eventos adversos (ocorridos em 7 e 30 dias) com necessidade de atendimento médico e/ou hospitalização.

### Coleta e gerenciamento de dados

Os dados serão coletados através de uma ficha clínica ( *electronic case report form* , eCRF) disponível on-line e serão inseridos na eCRF por cada centro participante. O treinamento e o suporte para a utilização do sistema serão disponibilizados aos pesquisadores pelo centro coordenador.

Os dados serão coletados diretamente do paciente e/ou da família. Diversos procedimentos serão aplicados para garantir a qualidade dos dados ( Tabela 3 ). Os dados a serem coletados durante as visitas incluem:

**Tabela 3:** Passos para garantir a qualidade da coleta e do gerenciamento dos dados

Item	Definição
Visita de iniciação	Todos os pesquisadores participarão de uma visita de iniciação do centro (treinamento on-line) antes do início do estudo para garantir a consistência dos procedimentos, incluindo a coleta de dados.
Contato	Os pesquisadores poderão ligar para o Centro Coordenador do Estudo para resolver questões ou problemas que possam surgir.
Limpeza dos dados	Será realizada limpeza periódica dos dados para identificar inconsistências (aproximadamente a cada 15 dias). Os centros serão notificados de quaisquer inconsistências para fornecer correções.
Validação estatística	Técnicas estatísticas para a identificação de erros serão realizadas durante o estudo. Essas análises incluirão a identificação de dados ausentes, dados inconsistentes, desvios de protocolo, eventos adversos relatados incorretamente (dados considerados inconsistentes com a revisão médica centralizada) e avaliação sistemática de erros.
Revisão dos dados	O Centro Coordenador/Patrocinador irá revisar os relatórios detalhados sobre triagem, inclusão, seguimento, consistência e total preenchimento dos dados mensalmente e tomará medidas imediatas para resolver quaisquer problemas.

**Figura 2:**
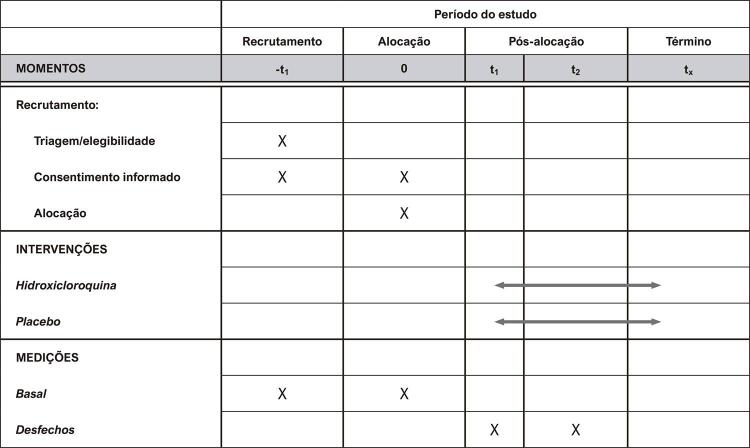
Esquema de coleta de dados e seguimento dos participantes.

1. Admissão (início do estudo):
  - Idade, sexo, estado civil, etnia, nível educacional, renda familiar e comorbidades;
  - Resultados de testes moleculares ou sorológicos para covid-19 (dependendo do tempo desde o início dos sintomas/diagnóstico clínico);
  - Uso concomitante de medicamentos no início do estudo;
  - Duração dos sintomas;
2. No sétimo dia após a randomização:
  - Avaliação da segurança (monitoramento de eventos adversos);
  - Adesão ao medicamento;
3. No 30º dia após a randomização:
  - Avaliação de eficácia (necessidade de hospitalização);
  - Avaliação de segurança (monitoramento de eventos adversos);

O esquema da coleta de dados e do seguimento dos participantes é apresentado na Figura 2 .

### Análise estatística

#### Cálculo amostral

Presume-se que o desfecho primário (hospitalização) ocorrerá em 20% dos indivíduos do grupo placebo e 14% dos indivíduos do grupo HCQ, correspondendo a uma redução de 30% no risco relativo. Essa premissa foi baseada na experiência clínica local nos primeiros meses da pandemia, em que 20% dos pacientes que não receberam nenhuma intervenção foram hospitalizados após o atendimento médico inicial. A escolha do efeito do tratamento (redução de 30% no risco relativo) baseou-se em plausibilidade razoável, com a maioria dos benefícios consistindo em efeitos moderados de tratamento (20-30%). Estimou-se que uma amostra de 1.230 pacientes (615 por grupo) forneceria um poder estatístico de 80% para detectar essa redução a um nível de significância estatística de 5% com o teste do qui-quadrado, premissa de hipótese de significância bilateral e alocação de 1:1. Foi estimada uma taxa de desistência de 5% em cada grupo, o que resultaria em 1.296 indivíduos no total (648 por grupo). Portanto, decidiu-se que 1.300 indivíduos (650 por grupo) seriam randomizados para o estudo. O cálculo amostral foi realizado no software SAS 9.4 (procedimento PROC POWER).^16 , 17^

#### Populações do estudo

A avaliação do desfecho primário (hospitalização em 30 dias) será realizada de acordo com o princípio da intenção de tratar ( *intention-to-treat* , ITT), que consistirá em todos os casos randomizados. Uma análise por ITT modificada (mITT) também será realizada após a exclusão de casos com teste negativo para covid-19.

#### Estatísticas descritivas gerais

Serão realizadas análises estatísticas após a resolução de todas as inconsistências, o controle de qualidade de dados e o bloqueio do banco de dados. As características clínicas e demográficas basais serão expressas em contagem e porcentagem, média e desvio padrão ou mediana e intervalo interquartil, conforme o caso. As análises de segurança irão considerar os desfechos de segurança, e os participantes serão classificados em grupos de tratamento de acordo com o medicamento que de fato receberam. Os motivos para a interrupção do estudo serão listados para cada grupo de tratamento.

A incidência de eventos adversos será resumida e apresentada por grupo de tratamento. Os eventos adversos serão resumidos de acordo com a gravidade e a intensidade por grupo de tratamento. Eventos adversos graves ou que levem à interrupção do tratamento serão listados por participante. O teste do qui-quadrado ou o teste exato de Fisher será utilizado para comparar os eventos adversos entre os dois grupos.

#### Análise do desfecho primário

O efeito da intervenção no desfecho primário e nos desfechos secundários binários será estimado por razão de risco (RR) e intervalo de confiança de 95% (IC95%). O teste do qui-quadrado ou o teste exato de Fisher será utilizado para os testes de hipótese. O mesmo procedimento será realizado para os desfechos secundários definidos por proporções.

O desfecho primário também será avaliado por meio de um modelo de regressão logística mista composto por um modelo de efeitos mistos com o grupo randomizado como efeito fixo e o centro como efeito aleatório. Será relatada a razão de chance com o IC95%.

#### Análise dos desfechos secundários

Os desfechos secundários definidos por variáveis quantitativas de distribuição assimétrica e normal serão comparados entre os dois grupos (HCQ e placebo) através do teste *t* de Student não pareado. O teste de Mann-Whitney será utilizado para as variáveis com distribuição não normal.

O modelo de regressão de Cox será utilizado para avaliar o efeito da intervenção na mortalidade aos 30 dias. Caso ocorra o fenômeno de probabilidade monótona, ou seja, observado um número raro de eventos, será aplicada a abordagem de probabilidade parcial penalizada de Firth no modelo de regressão de Cox univariado.

O modelo de regressão de Cox univariado será utilizado para avaliar o efeito da intervenção na sobrevida livre de hospitalizações aos 30 dias. A sobrevida livre de hospitalizações aos 30 dias será construída com o método de Kaplan-Meier, e o teste de log-rank será utilizado para avaliar as diferenças entre as curvas. Será relatada a razão de risco com o IC95%.

As hipóteses de riscos proporcionais serão verificadas através de somas cumulativas de resíduos de Martingale e o teste supremo do tipo Kolmogorov com base em uma amostra de 1.000 padrões de resíduos simulados.^18^

#### Análises interinas

Três análises interinas serão realizadas utilizando a abordagem de Haybittle-Peto para avaliar a segurança e a eficácia do estudo, especificamente quando o tamanho amostral atingir 25% (325 participantes), 50% (650 participantes) e 75% (975 participantes).^19^ A segurança será avaliada nos dias 7 e 30, e a eficácia, no dia 30, em blocos separados. Quanto à regra de decisão, na avaliação de segurança, o estudo pode ser interrompido, de acordo com o método de Haybittle-Peto, caso haja sinal de dano (arritmia cardíaca grave, morte súbita, retinopatia em 7 dias) com p < 0,01 (em cada análise interina). A porcentagem de pacientes a serem analisados na avaliação de segurança no dia 7 é de 25%, correspondendo a 325 participantes. Conforme descrito anteriormente, prevê-se a análise interina nesse momento. Na avaliação de eficácia, o estudo pode ser interrompido, de acordo com o método Haybittle-Peto, caso haja sinal de benefício (desfecho primário em 30 dias após a randomização) com p < 0,001 (em cada análise interina).

O limite de Haybittle-Peto é uma regra de interrupção conservadora na análise interina que possui um impacto mínimo no aumento de erros tipo I em ensaios com dois braços.^20^ Não haverá ajustes no limite final para significância estatística para análise sequencial.

Prevê-se que, durante as três análises interinas, a proporção entre casos negativos confirmados e aqueles que não foram testados será avaliada para estimar a necessidade de recálculo da amostra para garantir um poder estatístico adequado. De acordo com a proporção de casos não positivos de covid-19, será considerada a substituição da amostra, de forma a garantir um poder estatístico de 80% na amostra efetivamente analisada para o desfecho primário.

As análises serão realizadas a partir de dados completos. Além disso, será relatada a proporção de indivíduos não testados em cada grupo.

Todas as análises interinas pré-especificadas foram realizadas por um DSMB independente, que recomendou a continuação do estudo conforme o planejado após a realização de análises formais confidenciais e o envio de cartas oficiais ao Comitê Diretor do estudo COPE.

#### Sensibilidade e análise de subgrupos

As análises exploratórias para o desfecho primário serão realizadas considerando o efeito da intervenção dentro de subgrupos pré-especificados através de análises estratificadas e testes de interação. Esses testes de interação serão baseados em modelos de regressão logística binária que incluem o efeito do tratamento, o fator de interesse e um termo de interação entre as duas variáveis, com relato do valor de p para o termo de interação.

As análises serão conduzidas pelo princípio de análise de casos completos. Além disso, para o desfecho primário, se a proporção de dados ausentes for maior que 5%, uma análise de sensibilidade será realizada através de técnica de imputação de dados múltiplos.^21 , 22^

Todos os testes de hipótese serão bilaterais. Um valor de p < 0,05 será considerado estatisticamente significativo em todas as análises. As análises serão realizadas no *software* SAS, versão 9.4 (SAS Institute Inc, Cary, NC, EUA).

#### Bloqueio da base de dados

Será realizado o bloqueio da base de dados após a conclusão do seguimento de 30 dias de todos os pacientes. A limpeza dos dados será feita após o monitoramento clínico. Todas as análises interinas serão disponibilizadas às agências reguladoras locais do Brasil. Será concedido acesso à base de dados apenas aos membros do Comitê Diretor e aos estatísticos antes da publicação dos resultados principais.

#### Supervisão do ensaio clínico

O Comitê Executivo/Diretor é responsável pela supervisão geral do estudo, pelo desenvolvimento do protocolo de estudo e pela redação do manuscrito. Todos os demais comitês respondem ao Comitê Executivo/Diretor.

O DSMB irá avaliar os efeitos da HCQ comparada ao cuidado usual em relação ao desfecho primário (hospitalização em 30 dias) e à ocorrência de eventos adversos (em 7 e 30 dias) com necessidade de atendimento médico e/ou hospitalização. As regras para a interrupção precoce do estudo serão aplicadas ao objetivo primário (eficácia) e aos eventos adversos (segurança). O Comitê irá monitorar qualquer evento adverso grave e, se necessário, recomendar a interrupção do tratamento para garantir a segurança do paciente.

## Ética e disseminação

Os registros dos participantes serão mantidos em sigilo e acessados de forma restrita apenas por indivíduos formalmente ligados ao estudo, que irão transferir as informações clínicas para formulários específicos (que não contêm informações que possam identificar os pacientes) e verificar se o estudo está sendo conduzido de forma adequada. O formulário eletrônico de coleta de dados incluirá a ID do participante e a ID do centro correspondente. Após a explicação completa dos riscos/benefícios e dos procedimentos do estudo, todos os pacientes ou representantes legais devem assinar um TCLE em conformidade com as exigências locais.

O estudo foi aprovado pela Comissão Nacional de Ética em Pesquisa (CONEP) e pela Agência Nacional de Vigilância Sanitária (ANVISA). Está registrado no Registro Brasileiro de Ensaios Clínicos (REBEC) sob o número RBR-3cbs3w e no clinictrial.gov sob o código NCT04466540. Quaisquer alterações feitas no protocolo devem ser aprovadas pelo comitê de ética em pesquisa/sistema CONEP antes da implementação pelos centros participantes.

O pesquisador e a equipe do centro conduzirão o estudo de acordo com os princípios da Declaração de Helsinki, seguindo os princípios da boa prática clínica e todas as políticas e procedimentos regulatórios e internos aplicáveis.

Após a conclusão, o estudo será submetido para publicação independentemente dos resultados e será divulgado conforme solicitado pelas autoridades locais.

## * Material suplementar

Para informação adicional, por favor, clique aqui .
