# Supplementary material for: Justificativa e Desenho do Ensaio Clínico Randomizado COVID-19 Outpatient Prevention Evaluation (COPE - Coalition V): Hidroxicloroquina vs. Placebo em Pacientes Não Hospitalizados
Source: Arq Bras Cardiol. 2022 Feb 14;118(2):378–87. [Article in Portuguese] doi: 10.36660/abc.20210832 (PMC8856682; doi:10.36660/abc.20210832)
Supplement: Translation

# Translation: Rationale and Design of the COVID-19 Outpatient Prevention Evaluation (COPE - Coalition V) Randomized Clinical Trial: Hydroxychloroquine vs. Placebo in Non-Hospitalized Patients

**Authors:** Haliton Alves de Oliveira, Cleusa P. Ferri, Icaro Boszczowski, Gustavo B. F. Oliveira, Alexandre B. Cavalcanti, Regis G. Rosa, Renato D. Lopes, Luciano C. P. Azevedo, Viviane C. Veiga, Otavio Berwanger, Álvaro Avezum

**Affiliations:** 1 Brazil Centro Internacional de Pesquisa, Hospital Alemão Oswaldo Cruz, São Paulo, SP – Brazil; 2 Brazil Instituto de Pesquisa HCor, São Paulo, SP – Brazil; 3 Brazil Brazilian Research in Intensive Care Network (BRICnet), São Paulo, SP – Brazil; 4 Brazil Hospital Moinhos de Vento, Porto Alegre, PR – Brazil; 5 Brazil Brazilian Clinical Research Institute (BCRI), São Paulo, SP – Brazil; 6 USA Duke University Medical Center – Duke Clinical Research Institute, Durham – USA; 7 Brazil Instituto de Ensino e Pesquisa do Hospital Sírio-Libanês, São Paulo, SP – Brazil; 8 Brazil Beneficência Portuguesa de São Paulo, São Paulo, SP – Brazil; 9 Brazil Academic Research Organization, Hospital Israelita Albert Einstein, São Paulo, SP – Brazil

**Keywords:** COVID-19, SARS-CoV-2, Hydroxychloroquine, Randomized Controlled Trial

## Abstract

**Background:**

Despite the need for targeting specific therapeutic options for coronavirus disease 2019 (COVID-19), there has been no evidence of effectiveness of any specific treatment for the outpatient clinical setting. There are few randomized studies evaluating hydroxychloroquine (HCQ) in non-hospitalized patients. These studies indicate no benefit from the use of HCQ, but they assessed different primary outcomes and presented important biases for outcome evaluation.

**Objective:**

To evaluate if HCQ may prevent hospitalization due to COVID-19 compared to a matching placebo.

**Methods:**

The COVID-19 Outpatient Prevention Evaluation (COPE) study is a pragmatic, randomized, double-blind, placebo-controlled clinical trial evaluating the use of HCQ (800 mg on day 1 and 400 mg from day 2 to day 7) or matching placebo for the prevention of hospitalization due to COVID-19 in early non-hospitalized confirmed or suspected cases. Inclusion criteria are adults (≥ 18 years) seeking medical care with mild symptoms of COVID-19, with randomization ≤ 7 days after symptom onset, without indication of hospitalization at study screening, and with at least one risk factor for complication (> 65 years; hypertension; diabetes mellitus; asthma; chronic obstructive pulmonary disease or other chronic lung diseases; smoking; immunosuppression; or obesity). All hypothesis tests will be two-sided. A p-value < 0.05 will be considered statistically significant in all analyses. Clinicaltrials.gov: NCT04466540.

**Results:**

Clinical outcomes will be centrally adjudicated by an independent clinical event committee blinded to the assigned treatment groups. The primary efficacy endpoint will be assessed following the intention-to-treat principle.

**Conclusion:**

This study has the potential to reliably answer the scientific question of HCQ use in outpatients with COVID-19. To our knowledge, this is the largest trial evaluating HCQ in non-hospitalized individuals with COVID-19.

## Introduction

In December 2019, a group of patients with pneumonia of unknown cause was identified in Wuhan, in the Hubei province, China.^1^ High-Throughput sequencing from lower respiratory tract samples indicated a novel coronavirus, named 2019 novel coronavirus (2019-nCoV) or, more recently, severe acute respiratory syndrome coronavirus 2 (SARS-CoV-2), causing a complicated clinical condition affecting lung function (named coronavirus disease 2019, or COVID-19), which had not been previously detected in humans or animals.^1^

Despite the need for specific therapeutic options for COVID-19, there is no clear evidence of effectiveness for any treatment in the outpatient setting. Therefore, it is essential to evaluate therapeutic options such as pharmacological agents with antiviral effects to reduce the risk of clinical deterioration, hospitalization, mechanical ventilation requirement, and death, specifically in an early phase of COVID-19 in the outpatient setting. Currently, there are some randomized controlled trials (RCTs) evaluating chloroquine/hydroxychloroquine (HCQ) in non-hospitalized patients. In the context of pre/post-exposure prophylaxis, clinical studies have indicated a lack of benefit in terms of COVID-19 infection rate^5^ while others have found a higher occurrence of adverse events in patients receiving chloroquine/HCQ.^6 , 7^ Nevertheless, it is worth mentioning that those studies presented important biases and, when considered together, they have significant heterogeneity in the results due to different dosing regimens, inclusion criteria, and primary endpoints.

Considering non-hospitalized COVID-19 cases, RCTs have found no significant difference in hospitalization rate when HCQ was compared either to placebo^10^ or usual care.^11^ Furthermore, some RCTs have revealed no benefit in virological cure or reduction in viral load when HCQ was compared either to placebo^12^ or usual care.^10^ Some trials have even reported an increased occurrence of adverse events in patients receiving HCQ.^10 , 11^ Therefore, larger studies with greater methodological rigor are needed.

The main objective of this trial is to assess whether early treatment with HCQ will decrease the risk of hospitalization (primary efficacy endpoint) due to a COVID-19-related clinical reason within 30 days of randomization.

## Methods

### Study Design

This is a pragmatic, multicenter, double-blind, randomized, placebo-controlled clinical trial with an allocation ratio of 1:1. The study will evaluate the potential antiviral effects of an early treatment with HCQ (800 mg PO on day 1 and 400 mg PO from day 2 to day 7) vs. matching placebo for the prevention of hospitalization due to COVID-19-related complications in non-hospitalized confirmed or suspected COVID-19 cases. The planned study workflow is shown in Figure 1 . This protocol follows the Standard Protocol Items: Recommendations for Interventional Trials (SPIRIT) statement (Supplementary Table 1).

**Figure 1:** CONSORT diagram showing the study workflow and planned recruitment.

### Study Setting

The study will be conducted in 56 centers across the Brazilian geographical regions. Centers are both private and public outpatient health care services which have been approved to participate after favorable feasibility assessment, compliance with good clinical practices, and ethics approval.

### Primary Objective

We aim to assess whether treatment with HCQ is associated with reduced requirement for hospitalization due to a clinical reason related to confirmed or suspected COVID-19 within 30 days of randomization in the outpatient setting. Hospitalization is defined as hospital stay for a period ≥ 24h or at least one additional day of adjudicated hospitalization. The hospitalization criteria will follow local clinical practice of each participating site.

### Secondary Objectives

To assess the effect of treatment with HCQ compared to placebo in outpatients with suspected or confirmed COVID-19 on the following outcomes at 30 days of follow-up:

1. Uncontrolled asthma after ≥ 5 days of starting study medication: Affirmative answer in three or four items of the Global Initiative for Asthma (GINA) questionnaire, described in Supplementary Table 2;
2. Pneumonia: Defined by clinical-radiological criteria, which include a history of cough and one or more of the following signs/symptoms: sputum, dyspnea, chest pain, sweating or fever (> 37.8o C) + chest computed tomography scan showing unilateral or bilateral ground-glass opacity, focal consolidations, or mixed opacities (including reverse halo sign);
3. Otitis media: Defined by clinical criteria of fever (> 37.8o C) and otalgia + bulging of the tympanic membrane;
4. Fever resolution time: Day 0 of fever resolution will be defined as the first afebrile day (< 37.5o C) after enrolment into the study followed by at least two consecutive days. Temperature will be obtained through participant report in the patient’s diary;
5. Time to improvement of respiratory symptoms (cough, runny nose);
6. Hospitalization in an intensive care unit (ICU): Admission to ICU due to clinical reasons related to COVID-19;
7. Need for orotracheal intubation: Clinical need as assessed by the attending physician;
8. Mechanical ventilation time: Number of days on mechanical ventilation until extubation or death;
9. Mortality: Death due to any cause that occurs within 30 days of randomization;

We will also assess clinical safety within 30 days of randomization:

1. Hypoglycemia: Change in the frequency of hypoglycemic episodes in diabetic patients using hypoglycemic medication, perceived by clinical signs or symptoms or measured in a capillary or venous blood glucose device;
2. Palpitations: Patient perception of heartbeats that can possibly be diagnosed as arrhythmias in patients without known history of prolonged QTc interval or pre-existing heart disease;
3. Reduced visual acuity: Change in visual acuity or new diagnosis of retinal disease not previously documented;
4. Diarrhea: Change in bowel habit greater than three diarrheal episodes per day during HCQ use and three days after the end of treatment;
5. Anorexia: Change in appetite during HCQ use and three days after the end of treatment;
6. Emotional lability: Perception of change in emotional lability (mood swings) during HCQ use and three days after the end of treatment.

### Exploratory objectives

To assess the effect of treatment with HCQ compared to placebo in outpatients with suspected or confirmed COVID-19 on the following outcomes:

1. Time to hospitalization after randomization;
2. Assessment of patient’s clinical status at the time of hospitalization.

### Eligibility Criteria

COVID-19 case definition:

- The initial clinical evaluation of patients and their study eligibility screening will be assessed based on the classification of confirmed and suspected cases, developed according to the Brazilian Ministry of Health Guidelines and World Health Organization recommendations^13 , 14^ on the definition of cases, modified to the outpatient setting ( Table 1 ). Inclusion and exclusion criteria are described in Table 2 , including those related to cardiovascular safety as HCQ may increase the QTc interval.^15^
  **Table 1:** COVID-19 case definition for the COPE study (COALITION V) adapted according to Brazilian Ministry of Health and World Health Organization guidelines

COVID-19 status	Definition
Confirmed	Individual with laboratory confirmation of COVID-19 (RT-PCR SARS-CoV-2 virus detection), preferably collected between the 4th and 7th days of symptom onset by nasopharyngeal/oropharyngeal swabs, regardless of signs and symptoms.
	Immunological test (rapid test or classic serology to detect IgM/IgG antibodies) in a sample collected after the 7th day of symptom onset, analyzed by a validated method.
Suspected	Patient who meets at least one of the following criteria*:
	Patient with acute respiratory disease (fever AND at least one sign/symptom of respiratory disease, for example, cough or dyspnea) AND a travel history or residing in a location that reports community transmission of COVID-19 during the 14 days prior to the onset of symptoms;
	Patient with acute respiratory disease AND having been in contact with a confirmed or probable case (with acute respiratory disease without laboratory confirmation) of COVID-19 in the 14 days prior to the onset of symptoms.

* Depending on patient’s clinical status, these criteria can be complemented by radiological findings (interstitial infiltration in chest radiograph and/or ground-glass opacity in lung computed tomography scan). It should be noted that we will mostly treat patients with mild symptoms who do not have a clinical indication for an imaging test.
  **Table 2:** Inclusion and exclusion criteria

General Criteria	
	Adults (> 18 years) seeking medical care with suspected or confirmed COVID-19, with ≤ 7 days from symptom onset, presenting with mild symptoms without clear indication for hospitalization and at least one of the following risk factors for clinical complications:
**Inclusion Criteria**	
1.	Age > 65 years;
2.	Hypertension;
3.	Diabetes mellitus;
4.	Asthma;
5.	Chronic obstructive pulmonary disease or other chronic lung diseases;
6.	Smoking;
7.	Immunosuppression;
8.	Obesity (defined as body mass index ≥ 30 kg/m2).
**Exclusion Criteria**	
1.	Immediate hospitalization after first medical care;
2.	Positive test for influenza at the first medical care;
3.	Known hypersensitivity to hydroxychloroquine/chloroquine;
4.	Previous diagnosis of retinopathy or macular degeneration;
5.	Previous diagnosis of long QT syndrome, history of sudden death in close family members (parents and siblings), decompensated heart failure, unstable coronary artery disease, use of antiarrhythmic drugs or other drugs that can increase the bioavailability of hydroxychloroquine or enhance its effect;
6.	Evidence of known liver disease, reported by the patient;
7.	Evidence of known chronic kidney disease, reported by the patient;
8.	Patients with pancreatitis;
9.	Baseline electrocardiogram with QTc interval ≥ 480 ms;
10.	Chronic use of hydroxychloroquine/chloroquine for other reasons;
11.	Pregnancy.

### Randomization Method and Allocation Concealment

Randomization (1:1) will be generated by a web-based software and performed in permuted blocks of size 8. Concealment of the randomization list is maintained through a 24-hour, centralized, automated, internet-based randomization system.

### Blinding

Patients, investigators, and health care providers will be blinded to allocation of study drugs. Clinical outcomes will be assessed under blinded fashion by the Clinical Event Adjudication Committee.

### Trial Interventions

Both arms will receive usual care according to local practice, which basically comprises general advice and medications for symptom relief. Standard support and care measures are defined as any treatments other than the study medications that are necessary for the care of the patient with COVID-19, at the attending physician’s discretion.

Patients in the HCQ group will receive a dose of 400 mg BID on day 1 and a dose of 400 mg OD from day 2 to day 7. Patients in the matching placebo group will follow the same regimen of administration.

### Outcome Assessment and Follow-up

There will be two telephone contacts with the participants (7 and 30 days) to evaluate adherence, symptoms, and the need to seek medical attention in order to detect possible disease progression or adverse events resulting from the treatment.

### Treatment Interruption

In patients with suspected infection, the following procedure will be done if there is confirmation of negative COVID-19 tests:

- When testing for SARS-CoV-2 (reverse transcription polymerase chain reaction, RT-PCR) is performed at the hospital where the patient is randomized, the center’s principal investigator will obtain this information and send it to the coordinating center (Hospital Alemão Oswaldo Cruz International Research Center). The patient will be advised to stop treatment and will continue to be monitored until the end of follow-up at 30 days.
- When testing for SARS-Cov-2 (RT-PCR) is performed in another laboratory, the study participant will contact the center where the patient is randomized, which will then inform the coordinating center. The patient will be advised to stop treatment or not accordingly, with follow-up being continued for up to 30 days.

The study will be interrupted in case of clear benefit of the intervention for the primary outcome or in case of an increase in the frequency of serious adverse events. The Data and Safety Monitoring Board (DSMB) will closely monitor any occurrence of unforeseen adverse events and all serious adverse events to, if necessary, recommend the study end to ensure the safety of the patients.

### Adverse Event Report and Management

Adverse events are not considered to be a study endpoint in this protocol, except for adverse events classified as serious (hospitalization due to COVID-19 and death).

Adverse events will be actively collected from the moment the study participant signs the informed consent form. The information collected should include data from the patient’s clinical history and comorbidities, diagnosis of the event (based on signs and symptoms), classification of severity, start date, definition of the likelihood of causal relationship, as well as cause of the event according to the investigator, medical decision, patient evolution regarding adverse outcomes, criteria used to classify the severity of the event, and end date.

### Endpoint Reporting and Adjudication

Primary endpoint will be assessed by research physicians with previous and current experience in validating clinical events using international standards. Hospitalizations within 30 days due to COVID-19-related causes will be documented by the medical team. The information will be collected for analysis by the Clinical Event Adjudication Committee under confidential allocation (blinded fashion for clinical event assessment) following standardized criteria. The DSMB will assess the effects of HCQ compared to placebo treatment for the primary outcome measure (hospitalization within 30 days) and adverse events (occurring up to 7 and 30 days) requiring medical attention and/or hospitalization.

### Data Collection and Management

Data will be collected through an electronic case report form (eCRF) by Internet and will be entered into the eCRF by each participating site. Training and support for using the system will be made available to researchers by the coordinating center.

Data will be collected directly from the patient and/or family. We will apply several procedures to ensure data quality ( Table 3 ). Data to be collected during study visits include:

**Table 3:** Steps to ensure quality on data collection and management

Item	Definition
Initiation visit	All researchers will participate in a site initiation visit (web-based training) before the start of the study to ensure consistency of study procedures, including collection of the data.
Contact	Researchers will be able to call the Study Coordinating Center to solve issues or problems that may arise.
Data cleaning	Data cleaning to identify inconsistencies will be conducted periodically (approximately every 15 days). Centers will be notified about inconsistencies to provide correction.
Statistical validation	Statistical techniques for identifying errors will be carried out during the study. These analyses will include identification of missing data, inconsistent data, protocol deviations, adverse events reported incorrectly (data considered inconsistent with centralized medical review), and systematic error assessment.
Data review	The Coordinating/Sponsoring Center will review detailed reports monthly on screening, inclusion, follow-up, consistencies, and completeness of the data, and will take immediate action to solve any problems.

1. Admission (baseline):
  - Age, sex, marital status, ethnicity, educational level, family income, and comorbidities;
  - Results of molecular or serology tests for COVID-19 (depending on time from symptom onset/clinical diagnosis);
  - Use of concomitant medications at baseline;
  - Duration of symptoms;
2. At 7 days post-randomization:
  - Safety assessment (adverse event monitoring);
  - Medication adherence;
3. At 30 days post-randomization:
  - Efficacy assessment (need for hospitalization);
  - Safety assessment (adverse event monitoring);

The scheme for data collection and participant follow-up is shown in Figure 2 .

**Figure 2:** Data gathering and participant follow-up scheme.

### Statistical Analysis

#### Sample Size Calculation

We will assume that the primary outcome (hospitalization) will occur in 20% of individuals in the placebo group and 14% of individuals in the HCQ group, which corresponds to a relative risk reduction of 30%. This assumption was based on initial clinical experience locally in the first months of the pandemic, in which 20% of individuals without any intervention were hospitalized after initial medical care. The choice of treatment effect (a relative risk reduction of 30%) was based on reasonable plausibility, with most benefits consisting of moderate treatment effects (20-30%). It was estimated that a sample of 1230 individuals (615 per group) would provide 80% statistical power to detect this reduction at a significance level of 5% using the chi-square test, assuming a two-sided significance hypothesis, and considering a 1:1 allocation. The estimated dropout rate was 5% in each group, which would result in 1296 individuals (648 per group). Therefore, we decided that 1300 individuals (650 per group) would be randomized into the study. The sample size was calculated using SAS 9.4 (PROC POWER procedure).^16 , 17^

#### Study Populations

The assessment of the primary outcome (hospitalization within 30 days) will be performed following the intention-to-treat (ITT) principle, which will consist of all randomized cases. A modified intention-to-treat (mITT) analysis will also be performed after exclusion of COVID-19 negative-tested cases.

#### General Descriptive Statistics

Statistical analyses will be performed after resolving all inconsistencies, making data quality control, and locking the database. Baseline demographic and clinical characteristics will be expressed as count and percentage, mean and standard deviation, or median and interquartile range, whenever appropriate. Safety analyses will consider the safety outcomes, and the study participants will be classified in treatment groups according to the medication they actually used. The reasons for discontinuing the study will be listed for each treatment group.

The incidence of adverse events will be summarized and presented by treatment group. Adverse events will be summarized according to severity and intensity by treatment group. Serious adverse events or events that lead to treatment interruption will be listed by study participant. A comparison of adverse events between the two treatment groups will be performed using chi-square test or Fisher exact test.

#### Primary Outcome Analysis

The effect of the intervention on the primary outcome and on binary secondary outcomes will be estimated with risk ratio (RR) and 95% confidence interval (CI). Chi-square test or Fisher exact test will be employed for hypothesis testing. The same will be done for the secondary outcomes defined by proportions.

Primary outcome will be also assessed with a mixed logistic regression model using a mixed-effects model with randomized group as fixed effect and site as random effect. Odds ratio with 95% CI will be reported.

#### Secondary Outcome Analysis

Secondary outcomes defined by quantitative variables of normal and asymmetric distribution will be compared between the two groups (HCQ vs. placebo) using unpaired Student t-test or Mann-Whitney test for non-normally distributed variables.

The effect of the intervention on mortality at 30 days will be evaluated using the Cox regression model. If the phenomenon of monotone likelihood occurs or a rare number of events is observed, Firth penalized partial likelihood approach in a univariate Cox regression model will be applied.

The effect of intervention on hospitalization-free survival at 30 days will be assessed by applying the univariate Cox regression model. Hospitalization-free survival at 30 days will be constructed using the Kaplan-Meier method, and the log-rank test will be used to assess differences between curves. Hazard ratio with 95% CI will be reported.

Proportional hazard assumptions will be checked using cumulative sums of Martingale residuals and a Kolmogorov-type supremum test based on a sample of 1000 simulated residual patterns^18^ .

#### Interim Analyses

Three interim analyses to assess safety and effectiveness wil be performed when the sample size reaches 25% (325 individuals), 50% (650 individuals), and 75% (975 individuals) using the Haybittle–Peto approach.^19^ Safety will be assessed at 7 and 30 days, and effectiveness at 30 days, in separate blocks. As for the decision rule, in the safety assessment, the study may be interrupted, according to the Haybittle–Peto method, if there is a sign of harm (severe cardiac arrhythmia, sudden death, retinopathy at 7 days) with a p < 0.01 (in each interim analysis). The percentage of patients to be analyzed in the safety assessment at 7 days is 25%, which corresponds to 325 individuals. The first interim analysis is foreseen at this point, as described above. In the evaluation of effectiveness, the study may be interrupted, according to the Haybittle–Peto method, if there is a sign of benefit (primary outcome within 30 days of randomization) with a p < 0.001 (in each interim analysis).

The Haybittle–Peto boundary is a conservative stopping rule at interim analysis that has minimal impact on increasing type I error in two-arm trials.^20^ There will be no adjustments in the final threshold for statistical significance for sequential analysis.

We predict that, during three interim analyses, the proportion of confirmed negative-tested cases and those who have not been tested will be evaluated to estimate the need for sample recalculation to ensure adequate statistical power. According to the proportion of non-positive COVID-19 cases, sampling replacement will be considered in order to guarantee 80% statistical power in the sample effectively analyzed for the primary endpoint.

Analyses will be conducted using complete data. Additionally, the proportion of untested individuals in each group will be reported.

All of the prespecified interim analyses were carried out by an independent DSMB, which recommended the study continuation as planned after formal confidential reviews and official letters to the COPE Study Steering Committee.

#### Sensitivity and Subgroup Analysis

Exploratory analyses for the primary outcome will be done considering effect of the intervention within prespecified subgroups using stratified analyses and interaction tests. These interaction tests will be based on binary logistic regression models that include the treatment effect, the factor of interest, and an interaction term between the two variables, with reporting of the p-value for the interaction term.

Analyses will be conducted using the complete-case analysis principle. Additionally, for the primary outcome, if the proportion of missing data is greater than 5%, a sensitivity analysis will be conducted using a multiple data imputation technique.^21 , 22^

All hypothesis tests will be two-sided. A p-value < 0.05 will be considered statistically significant in all analyses. Analyses will be done using SAS, version 9.4 (SAS Institute Inc, Cary, NC, USA).

#### Database Lock

Database lock will be carried out after completion of the 30-day follow-up for all patients, and data cleaning will be done after clinical monitoring. All interim analyses will be made available to local regulatory agencies in Brazil. Database access will be granted only to the Steering Committee members and statisticians before the main results are published.

#### Trial Oversight

The Executive/Steering Committee is responsible for the general supervision of the study, development of the study protocol, and manuscript writing. All other study committees report to the Executive/Steering Committee.

The DSMB will assess the effects of HCQ compared to usual care on the primary endpoint (hospitalization within 30 days) and adverse events (within 7 and 30 days) requiring medical care and/or hospitalization. The rules for early discontinuation of the trial will be applied to the primary objective (efficacy) as well as to serious adverse events (safety). The Committee will monitor any serious adverse event and recommend treatment interruption, if necessary, to ensure patient safety.

## Ethics and Dissemination

Records of all study participants will be kept confidential and will be accessed in a restricted way only by people formally related to the study, who will transfer the clinical information to specific forms (which do not contain information that can identify individuals) and verify whether the study is being conducted properly. The electronic data collection form will contain the patient study ID and the corresponding site ID. Each study patient or legal representative must provide written consent in accordance with local requirements, after the risks/benefits and study procedures have been fully explained.

This study was approved by the Brazilian National Research Ethics Commission (CONEP) and National Health Surveillance Agency (ANVISA). It is registered with the Brazilian Clinical Trial Network (REBEC) with registration number RBR-3cbs3w and in clinicaltrial.gov with identification number NCT04466540. All amendments to the protocol must be approved by the institutional review board/CONEP system before implementation by the participating sites.

The site investigator and study team will conduct the study in accordance with the principles of the Helsinki Declaration, following the principles of good clinical practice as well as all applicable regulatory and internal policies and procedures.

This study will be submitted for publication regardless of its results after completion. It will also be disseminated as requested by local authorities.

## * Supplemental Materials

For additional information, please click here.
